# Cost-Effectiveness of Artificial Intelligence Support in Computed Tomography-Based Lung Cancer Screening

**DOI:** 10.3390/cancers14071729

**Published:** 2022-03-29

**Authors:** Sebastian Ziegelmayer, Markus Graf, Marcus Makowski, Joshua Gawlitza, Felix Gassert

**Affiliations:** Institute of Diagnostic and Interventional Radiology, School of Medicine, Klinikum Rechts der Isar, Technical University Munich, Ismaninger Straße 22, 81675 Munich, Germany; markus.m.graf@tum.de (M.G.); marcus.makowski@tum.de (M.M.); joshua.gawlitza@tum.de (J.G.); felix.gassert@tum.de (F.G.)

**Keywords:** lung cancer screening, deep learning, cost-effectiveness analysis, AI-support system

## Abstract

**Simple Summary:**

Lung cancer screening with low-dose CT (LDCT) has been shown to significantly reduce cancer-related mortality and is recommended by the United States Preventive Services Task Force (USPSTF). With pending recommendation in Europe and millions of patients enrolling in the program, deep learning algorithms could reduce the number of false positive and negative findings. Therefore, we evaluated the cost-effectiveness of using an AI algorithm for the initial screening scan using a Markov simulation. We found that AI support at initial screening is a cost-effective strategy up to a cost of USD 1240 per patient screening, given a willingness-to-pay of USD 100,000 per quality-adjusted life years (QALYs).

**Abstract:**

Background: Lung cancer screening is already implemented in the USA and strongly recommended by European Radiological and Thoracic societies as well. Upon implementation, the total number of thoracic computed tomographies (CT) is likely to rise significantly. As shown in previous studies, modern artificial intelligence-based algorithms are on-par or even exceed radiologist’s performance in lung nodule detection and classification. Therefore, the aim of this study was to evaluate the cost-effectiveness of an AI-based system in the context of baseline lung cancer screening. Methods: In this retrospective study, a decision model based on Markov simulation was developed to estimate the quality-adjusted life-years (QALYs) and lifetime costs of the diagnostic modalities. Literature research was performed to determine model input parameters. Model uncertainty and possible costs of the AI-system were assessed using deterministic and probabilistic sensitivity analysis. Results: In the base case scenario CT + AI resulted in a negative incremental cost-effectiveness ratio (ICER) as compared to CT only, showing lower costs and higher effectiveness. Threshold analysis showed that the ICER remained negative up to a threshold of USD 68 for the AI support. The willingness-to-pay of USD 100,000 was crossed at a value of USD 1240. Deterministic and probabilistic sensitivity analysis showed model robustness for varying input parameters. Conclusion: Based on our results, the use of an AI-based system in the initial low-dose CT scan of lung cancer screening is a feasible diagnostic strategy from a cost-effectiveness perspective.

## 1. Introduction

Based on the findings of the national lung screening trial (NLST), in 2014 the United States Preventive Service task force recommended the annual lung cancer screening of patients between 55 and 80 years with 20 pack years of smoking history [1,2]. In contrast to the high and further increasing incidence of lung cancer globally, the incidence of lung cancer was relatively low in the NLST. Nonetheless, the NLST was able to show a significant reduction in lung cancer related mortality due to the annual screening with low-dose computed tomography (CT). Consequently, a European Position Statement followed in 2017, strongly recommending the CT-based lung cancer screening as well [3]. This recommendation is further supported by the Dutch-Belgian lung-cancer screening trial (Nederlands-Leuvens Longkanker Screenings Onderzoek (NELSON)), which also showed a significant reduction in lung cancer mortality for high-risk patients who participated in the screening [4]. With several ongoing pilot projects in Europe, the widespread introduction of lung cancer screening seems to be only a matter of time.

Nevertheless, the benefits of lung cancer screening are limited by false negative and false positive findings, which not only result in high costs but also affect clinical outcome and quality of life [2,5,6]. Currently, low dose CT-scans in the screening setting are evaluated based on standardized systems like Lung-RADS (Lung imaging reporting and data system), which improve the diagnostic accuracy for radiologists and reduces costs by decreasing the need for further diagnostic tests [7,8]. Even after a recent revision of the reporting system, observer variability will remain a relevant limitation [9,10].

The rapid development of artificial intelligence (AI) in the medical field has shown promising results for cancer screening and recent AI-models may achieve or exceed the diagnostic performance of sub-specialized experts, for example in breast cancer screening [11]. While long-standing CAD (computer aided diagnosis/detection) systems show mixed results for lung cancer detection [12,13,14], novel neural networks, convolutional neural networks (CNN) in particular, seem to have a positive effect on the diagnostic performance of radiologists [15]. Ardila et al. showed that a 3D-CNN outperformed radiologists in low-dose CT screening scans when no prior scans were available, indicating a favorable benefit for screening initiation.

Among other constraints, the health economic impact of AI systems is an important factor in the decision to implement models in routine clinical practice. Despite the imminent deployment of lung cancer screening and the promising results of AI-systems, no study has been performed to evaluate the utilization of neural networks in lung cancer screening compared to the stand-alone low dose CT-scan from an economic point of view. Therefore, the aim of our study was to evaluate the cost effectiveness of an AI-system for the initial scan of annual lung cancer screening and present the first results on identifying a cost margin for a clinical integration.

## 2. Materials and Methods

### 2.1. Model Structure

**Figure 1:**
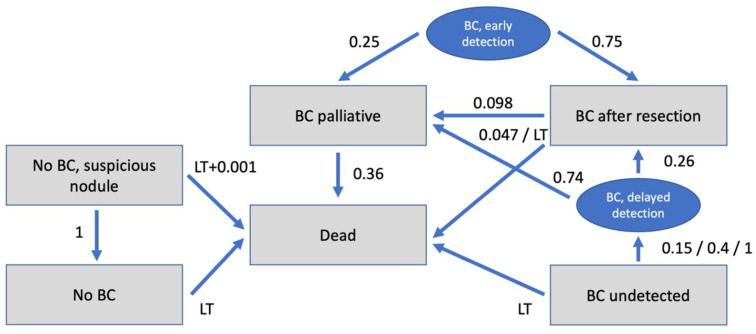
Markov model with possible states of disease and transition probabilities between states. BC = bronchial cancer; LT = life tables.

A decision model including the diagnostic strategies of conventional CT and CT augmented by AI was created and used as a decision tree, as shown in Figure 1.

For calculation of costs and benefits in the different iterations a Markov transition state model was created. The model included the stages:

- No BC (patients without BC = true negative);
- No BC, Suspicious nodule (patients without BC but suspicious nodule = false positive);
- BC undetected (patients with undetected BC = false negative);
- BC after resection (patients with BC after resection);
- BC palliative (patients with BC which is unresectable/palliative);
- Dead.

Additionally, for better simulation and understanding of the model, the states “BC delayed detection” and “BC early detection” were created, which only served for transition. The Markov model reflects the different states a patient can be assigned to. Taking into account transition probabilities between the states as well as costs and effectiveness (displayed in Quality of Life) in those states during several iterations, cumulative costs and cumulative effectiveness within a defined time horizon can be calculated by adding those up throughout the iterations.

Analysis of the model was performed using a dedicated decision analysis software (TreeAge Pro Version 19.1.1, Williamstown, MA, USA).

### 2.2. Input Parameters

There was no requirement for an ethical approval for this analysis based on commonly available data. Model input parameters were based on current literature. Age-specific risk of death was derived from the US life tables [16]. Age at the diagnostic procedure was set to 60 years and willingness-to-pay was set to USD 100,000 per quality adjusted life year (QALY) at a discount rate of 3%, as reported previously [17,18]. The discount rate reflects the loss in economic value or effectiveness when there is a delay in realizing a benefit or incurring costs. The pre-test probability of BC was set to 2.635% for the risk group consisting of female and male smokers risk for an interval of 30 years, according to published data from Jacob et al. [19]. All input parameters and corresponding references are listed in Table 1.

### 2.3. Diagnostic Test Performances

Sensitivity and specificity values for CT detection of BC with and without AI were derived from the literature (Table 1).

### 2.4. Costs

From a United States (US) healthcare perspective, costs were estimated based on Medicare data and available literature (Table 1). The long-term costs of the follow up in case of false positive was estimated at USD 2256 including the costs for a follow up CT examination and a possible bronchoscopy and biopsy [21]. The resection costs of BC were set to USD 36,305, according to Cowper et al. [22]. annual costs of palliative BC patients were estimated at USD 60,000 [21].

### 2.5. Utilities

Utility is measured in the additional quality-adjusted life years (QALY) which are gained through each diagnostic procedure. According to previous studies, quality of life (QOL) for curative BC patients was set to 0.79 for the first year after resection and 0.933 for the following years [24,25]. In accordance with the literature, QOL for palliative BC patients was set to 0.63 [26]. These values were then used for calculations in a Markov model specifically designed as mentioned above.

### 2.6. Transition Probabilities

Transition probabilities were derived from a systematic review of the recent literature and are shown in Table 1. Probability of successful resection of (early) detected BC was estimated at 75%, according to the national lung screening trial research team [2]. Risk of secondary occurrence of cancer/metastases after resection of the primary tumor was assumed to be 9.80% [29]. Annual mortality rate of curative patients was set to 4.7% and to 36.0% for palliative patients [28,32,33].

### 2.7. Cost-Effectiveness Analysis

The cost-effectiveness analysis was performed based on Markov simulations with a run time of 20 years (20 iterations) after initial diagnostic procedure. The discount rate was set to 3.0% and willingness-to-pay was set to USD 100,000 per QALY according to current recommendations [18].

In the base-case scenario, cost-effectiveness was determined with costs of CT + AI identical to costs of CT only, meaning costs of USD 0 for additional use of AI. Based on these results, maximum costs for AI were calculated for several willingness-to-pay thresholds. For evaluation of model uncertainty and influence of alteration of each variable on the model, a deterministic sensitivity analysis was performed. Results were visualized in a tornado diagram.

Based on the Markov model, Monte-Carlo simulations were used to perform a probabilistic sensitivity analysis with a total of 30,000 iterations. This method is used to account for the variation of input-parameters among different individuals.

## 3. Results

### 3.1. Cost-Effectiveness Analysis

**Figure 2:**
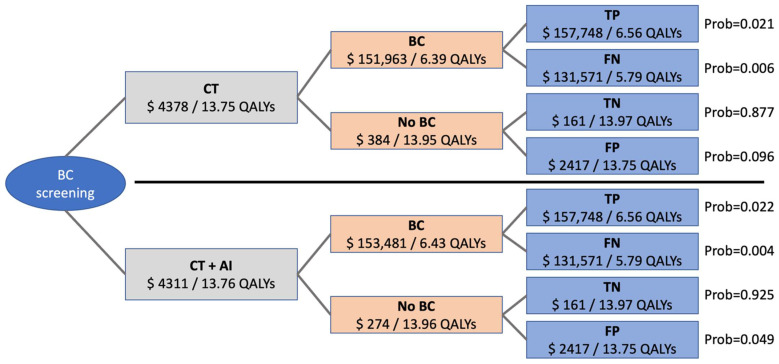
Roll-back of the economic model showing costs and effectiveness of the different outcomes. Distributions leading to overall costs and effectiveness are different for CT and CT + AI depending on sensitivity and specificity of the two methods and indicated as probabilities. BC = bronchial cancer; CT = computed tomography; TP = true positive; TN = true negative; FP = false positive; FN = false negative; Prob = probability.

Simulations of a time horizon of 20 years resulted in average cumulative costs of USD 4310.82 for CT + AI and USD 4378.44 for CT if additional diagnostic costs for the use of AI were set to USD 0 in the base case scenario. In this scenario, average cumulative effectiveness was at 13.76 QALYs for CT + AI and at 13.75 QALYs for CT. To better understand the impact of input parameters on the model, costs and effectiveness as well as distribution of the different outcomes are shown in Figure 2. Different overall costs and effectiveness derive from different distribution of the outcomes “true positive”, “false negative”, “true negative”, and “false positive” based on different sensitivity and specificity of the two methods. The incremental cost-effectiveness ratio in the base case scenario was negative, meaning both, lower cost and higher effectiveness for CT + AI.

### 3.2. Sensitivity Analysis

**Figure 3:**
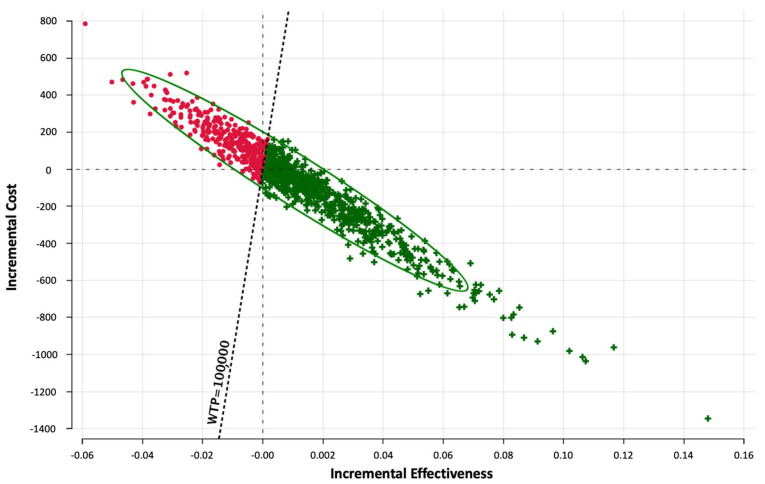
Probabilistic sensitivity analysis utilizing Monte-Carlo simulations (30,000 iterations). Incremental cost-effectiveness scatter plot for CT + AI vs. CT. iterations with an ICER-value below the willingness-to-pay of USD 100,000 per QALY are shown as green crosses. WTP = willingness-to-pay.

Probabilistic sensitivity analysis and Monte Carlo simulation was performed to determine the distribution of the resulting ICER-values and is visualized in Figure 3. Monte Carlo simulation reflects the difference between costs (=incremental costs) and effectiveness (=incremental effectiveness) for a certain amount of notional scenarios/iterations. All iterations with an ICER-value below the willingness-to-pay of USD 100,000 per QALY were considered cost-effective.

**Figure 4:**
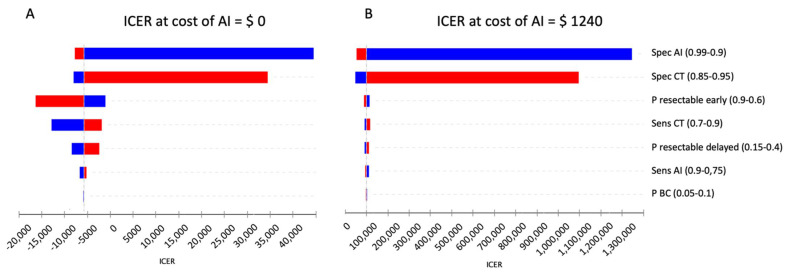
(**A**) Tornado diagram showing the impact of input parameters on incremental cost-effectiveness ratio (ICER) in the base case scenario. Assuming a willingness-to-pay threshold of USD 100,000 per QALY, CT + AI remained cost-effective in all cases. (**B**) Tornado diagram showing the impact of input parameters on incremental cost-effectiveness ratio (ICER) when costs of AI were set to USD 1240 with an expected value of USD 100,000 per QALY. Blue bars show changes when decreasing the value of an input parameter as compared to the base case scenario and red bars when increasing the respective value. Sens = sensitivity; Spec = specificity; CT = computed tomography; AI = artificial intelligence; P = probability.

Deterministic sensitivity analysis was performed to account for variability of input parameters in the base case scenario. Results are displayed as a tornado diagram in Figure 4A.

Applying wide ranges of variation for the different input parameters, ICER stayed below USD 0/QALY for the sensitivities of the diagnostic modalities and the probabilities of resectability in early and delayed diagnosis. Although ICER turned positive when varying the specificity of CT and CT + AI, the willingness-to-pay threshold of USD 100,000/QALY was not crossed in any of the cases.

### 3.3. Threshold Analysis

**Figure 5:**
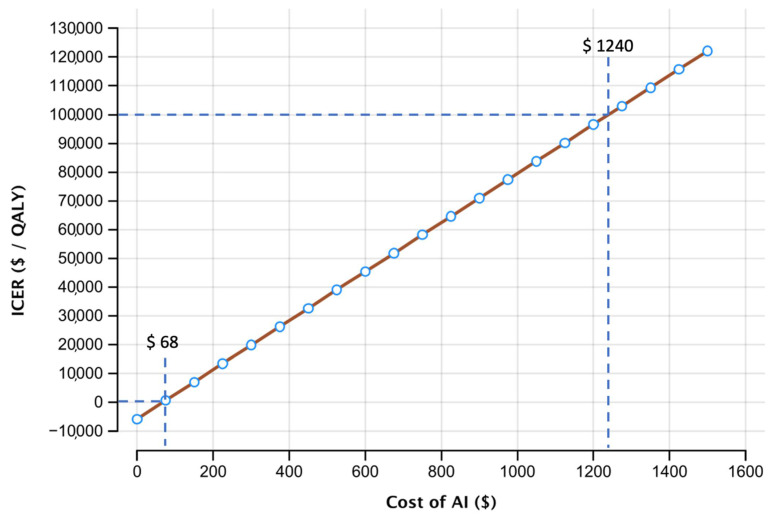
One-way sensitivity analysis for costs of AI (USD) and the corresponding incremental cost effectiveness ratio (ICER in USD/QALY). Thresholds indicate values at an ICER of USD 0/QALY and USD 100,000/QALY. ICER = incremental cost-effectiveness ratio; AI = artificial intelligence; QALY = quality adjusted life year.

To determine the maximum possible costs for the use of AI at a willingness-to-pay of USD 100,000/QALY, a threshold analysis was performed. As shown in Figure 5, ICER remained negative until costs of AI were raised to USD 68.

Raising costs of AI further, the assumed willingness-to-pay threshold of USD 100,000/QALY is only crossed at a value USD 1240. Influence in different input parameters in this second base case scenario setting costs of AI to USD 1240 are shown in Figure 4B. To account for possible variation of the willingness-to-pay, Table 2 displays possible costs for AI depending on different willingness-to-pay thresholds. Due to the cost’s dependency on the ICER, the cost for AI directly is further influenced by the systems performance, resulting in a higher price for a better system due to the increased ICER.

## 4. Discussion

The widespread integration of lung cancer screening is proving to be a complex and challenging undertaking. Nevertheless, lung cancer screening is a cost-effective method to reduce lung cancer mortality. AI-models for cancer detection and classification have proved to be of benefit in lung cancer screening in several studies [15,34].

In the present study, we show that a state-of-the-art AI-model (3D-convolutional neural network according to Ardila et al.) is a cost-effective method for the baseline screening scan [15]. Despite promising results of AI in the health care sector, studies evaluating the economic impact and cost effectiveness remain sparse [35]. To our knowledge, no study has been conducted to investigate the cost-effectiveness of an AI-system in lung cancer screening. Based on the superior performance of the AI-model without prior imaging, we simulated an implementation for the initial screening scan using input parameters derived from published screening cohorts [2,15,36,37], to ensure comparability to the standard screening setting.

Our base case estimate for screening with an AI system compared to current low-dose CT screening yielded a negative ICER up to costs of USD 68 for the AI system, indicating that using an AI system in the screening setting results in lower cost and higher effectiveness up to these costs per patient scan. Furthermore, the ICER remained below the applied willingness-to-pay up to costs of USD 1240. To account for variations in input parameters, we performed a deterministic sensitivity analysis for the base case scenario and the maximum cost-effective costs (USD 1240). The specificity of the diagnostic strategy had the greatest influence for both scenarios, due to the low lung cancer rate in screening cohorts. For the base case scenario all input variations resulted in an ICER below the willingness-to-pay by a large margin, indicating robust cost-effectiveness. Adding AI support showed a reduced number of false-positives and an increased number of true negatives in our simulation. In particular, the reduction of false-positives highly impacts the value of a screening method, as not only costs in the form of unnecessary follow-up examination and possibly further, partly invasive examinations are reduced, but also patients do not have to experience the psychological distress of a possible cancer diagnosis [38]. Additionally, the false positive rates and the frequency of invasive diagnostic procedures were more frequent at the baseline CT, ranging from 7.9% to 49.3% for the false positive rate and 3.7% for additional invasive procedures [2,39], further emphasizing the benefit of AI support for the initial screening. As shown by Audelan et al., the sensitivity and specificity of AI in lung cancer screening can further be improved, consequently allowing for an additional reduction of costs and increased effectiveness [40].

Despite promising results, our study underlies several limitations. First, the cost-effectiveness was only evaluated for the initial scan in the lung cancer screening. This is due to published literature, focusing on the superiority of AI lung nodule detection and classification in initial CT of the thorax without prior imaging for comparison. According to Ardila et al., deep-learning algorithms are superior to radiologists in lung cancer screening detection, when no prior imaging is available for comparison, but is on-par as soon as previous examinations are available for the reader. Consequently, further research has to be conducted to evaluate the cost-effectiveness of AI-based computer-aided diagnosis systems in longitudinal screening, beyond the initial scan [15]. Further, our evaluation is focused on the sole AI system performance in comparison to the human reader—the radiologist. However, several studies have shown promising results for the collaboration of both, often referred to as the “Centaur model” [33]. Such systems were shown not only to be beneficial in patient care but cost-effective as well [41]. Despite dealing with different challenges compared to lung cancer, for thyroid nodule detection, AI systems outperform thyroid cancer specialized radiologists in nodule classification, but the combination of specialized radiologists with AI-support showed an even higher specificity and positive predictive value when compared to the AI system alone [42]. Therefore, further research is needed to evaluate the combination of AI models and specialized thorax radiologists in lung cancer detection and its cost-effectiveness. Lastly, cost-effectiveness analysis with decision-based models is highly dependent on the input parameters, while deterministic sensitivity analysis may incorporate parameter variation to a certain degree, and recommendations for each individual case cannot be derived from the model.

## 5. Conclusions

To conclude, in our study we show that screening with an AI-model in the initial screening scan is a cost-effective strategy in low-dose CT lung cancer screening with robustness to variation of input parameters. Defining thresholds for cost of AI results might help faster translate AI systems into clinical use.

## Figures and Tables

**Table 1:** Input parameters.

Pre-test-Probability of BC	2.635	Jacob et al. [19]
Age at diagnostic procedure	60 years	US Preventive Services Task Force [1]
Assumed WTP	USD 100,000,00	Assumption
Discount rate	3.00%	Assumption
Markov model time	20 years	Assumption
Diagnostic Test Performances		
Sensitivity for BC CT	77.9%	Ardila et al. [15]
Specificity for BC CT	87.7%	Ardila et al. [15]
Sensitivity for BC CT + AI	97.7%	Ardila et al. [15]
Specificity for BC CT + AI	98.4%	Ardila et al. [15]
Costs (Acute)		
CT	USD 161.00	Medicare (71,250) [20]
Costs (Long Term)		
No BC	USD 0.00	
Follow up if false positive	USD 2256.00	ten Haaf et al. [21]
Curative therapy BC/resection cost	USD 36,305.00	Cowper et al. [22]
BC undetected	USD 0	Assumption
BC after resection	USD 4283.00	ten Haaf et al. [21]
Therapy BC, palliative	USD 60,000.00	ten Haaf et al. [21]
Dead	USD 0	Assumption
Utilities		
No BC	1	Assumption
Follow up if false positive	0.98	Gareen et al. [23]
Curative therapy BC/resection	0.79	Grutters et al. [24]
BC undetected	1	Assumption
BC after resection	0.933	Möller et al. [25]
BC palliative	0.63	Doyle et al. [26]
Dead	0	Assumption
Transition Probabilities		
Verification of suspicious nodule as no BC	100%	Assumption
Death if no BC but suspicious nodule	0.001 (invasive diagnostics) + life tables	The National Lung Screening Trial Research Team [2]
Resection rate of BC after early detection	75%	The National Lung Screening Trial Research Team [2]
Death after curative resection	4.70%	Green et al./Toker et al. [27,28]
Recurrence after resection	9.80%	Lou et al. [29]
Detection of initially undetected BC	15% 1st, 40% 2nd, 100% 3rd year	Scholten et al. [30]
Death with undetected BC	life tables	
Resection rate of BC after delayed detection	26%	Hunbogi et al. [31]
Death with palliative care	36%	Cancer Stat Facts: Lung and Bronchus Cancer, National Cancer Institute [32]
Death without BC	life tables	

AI = artificial intelligence; BC = bronchial cancer; CT = computed tomography; QALY = quality adjusted life year; WTP = willingness-to-pay.

**Table 2:** Cost of AI at different WTP-thresholds.

WTP (USD/QALY)	0	20,000	40,000	60,000	80,000	100,000	120,000	150,000	200,000
Cost of AI (USD)	68	302	537	771	1006	1240	1475	1826	2412

## Data Availability

The data that support the findings of this study are listed in Table 1.
